# Eliciting Willingness and Beliefs towards Participation in Genetic Psychiatric Testing in Black/African American Mothers at Risk for Depression

**DOI:** 10.3390/bs10120181

**Published:** 2020-11-26

**Authors:** Rahshida Atkins, Terri-Ann Kelly, Shanda Johnson, Wanda Williams, Yolanda Nelson, Paule V. Joseph, Deirdre Jackson, Deborah King, Tiffany Stellmacher, Nisoni-Davis Halty, Michelle Tinglin, Gale Gage

**Affiliations:** 1 School of Nursing, The College of New Jersey, Ewing, NJ 08628, USA; nelsony1@tcnj.edu (Y.N.); jacksond@tcnj.edu (D.J.); powelld1@tcnj.edu (D.K.); haltyn1@tcnj.edu (N.-D.H.); 2 School of Nursing, Rutgers the State University of New Jersey, Camden, NJ 08102, USA; tk503@camden.rutgers.edu (T.-A.K.); wanda.williams@rutgers.edu (W.W.); tcs98@scarletmail.rutgers.edu (T.S.); 3 Department of Nursing, New Jersey City University, Jersey City, NJ 07305, USA; sjohnson2@njcu.edu (S.J.); tinglinmichelle@gmail.com (M.T.); 4 National Institute of Alcohol Abuse and Alcoholism, National Institute of Nursing Research, Bethesda, MD 20892, USA; paule.joseph@nih.gov; 5 School of Nursing, Essex County College, Newark, NJ 07102, USA; gage@essex.edu or

**Keywords:** mental health disparities, genetic research, mental health, depressive symptoms, health disparities

## Abstract

Black/African American women are at high risk for depression, yet are underrepresented in psychiatric genetic research for depression prevention and treatment. Little is known about the factors that influence participation in genetic testing for Black/African American women at risk. The purpose of this study was to elicit the beliefs that underlie participation in genetic testing for depression in Black/African American mothers, a subgroup at high risk. Willingness to participate in genetic testing procedures was also determined. A qualitative, descriptive design was employed. Exactly 19 mothers aged 21–42 completed open-ended questionnaires. Directed content and descriptive analyses of the text were conducted based on the Theory of Planned Behavior. Salient beliefs included: behavioral advantages—diagnosing/detecting depression (31.6%), finding cure/treatment (21.1%); disadvantages—not finding follow-up treatment/help (21.1%); salient referents, who approves—family members (47.4%), agencies/organizations (26.3%); who disapproves—church associates (21.1%). Control beliefs included: barriers—unpleasant/difficult testing procedures (42.1%), limited knowledge about the purpose of testing (26.3%); facilitator—a convenient location (21.1%). Most mothers (89.5%) indicated willingness to participate in testing. Interventions can target families, address barriers, emphasize future benefits, and use convenient locations and community-based participatory research methods. Policies can address social determinants of participation to increase inclusion of these mothers in psychiatric genetic research.

## 1. Introduction

Clinical depression is a serious mental illness that affects 17.3 million (7.3%) United States (U.S.) adults each year and is more prevalent among females (8.7%) and younger adults (18–25, 13.1%) [1,2]. Although the prevalence of clinically diagnosed depression is higher among White/Caucasian Americans [1], Black/African Americans often report higher levels of depressive symptoms in national health surveys compared to other ethnic groups [3,4]. Disparities in regard to quality and access to mental health services contribute to more chronic and persistent depressive symptoms among Black/African Americans that often go undiagnosed and/or untreated with psychological counseling or medications, resulting in overall poorer mental health for these Americans [5,6,7,8]. 

These facts are reflected in what we know about Black/African American mothers, a subgroup of Black women at high risk for clinical depression. In published studies, between 50% and 75% of Black mothers report high levels of depressive symptoms that warrant a referral to determine if clinical depression is present [9,10,11,12]. This rate is up to six times the rate of clinical depression reported in the general population of the United States (U.S.) adult women (8.6%; [1]) and up to double the rate of depressive symptoms reported by Black women in general (21% to 39%; [13,14]). Black mothers also experience psychosocial and cultural stressors such as financial instability and poverty, anger, perceived racism and parenting stressors, violence, and complex relationship issues which increase their risk for clinical depression [9,12,15,16,17,18,19]. Black/African American mothers often do not receive nor seek professional psychological counseling or medical treatment for depressive symptoms, resulting in negative psychosocial and physical health consequences for these mothers and their children [10,16,17,18]. Mothers with both clinical depression and high levels of depressive symptoms are more likely to have poor health and engage in unhealthy behaviors [16]. Children of these mothers are also more likely to suffer from poor academic performance, behavioral problems, and delayed cognitive development, and are more likely to develop mental illness as adults [12,16,20,21]. Given these high rates of depressive symptoms, coupled with lack of treatment, preventive efforts are needed to combat depressive symptoms and avoid these deleterious effects in these mothers and their children. 

To support the development of preventive interventions and treatment for mental illness, the National Advisory Mental Health Council of the National Institutes of Mental Health recommended that scientists “make investments to capture genetic and phenotypic variation across diverse human populations” [22]. Disparities in mental health outcomes can be addressed in part through genetic research aimed at uncovering genetic causes of mental illness for select psychiatric conditions such as clinical depression [22,23,24]. Ongoing research has led to the identification of multiple genetic risk variants associated with clinical features of depression [25]. These basic scientific discoveries, although not yet conclusive regarding the exact genetic determinants of depression, have the potential to eventually inform depression prevention and treatment approaches as the quality of this scientific inquiry is enhanced [24,25]. However, racial/ethnic minority groups may not benefit from these scientific discoveries because they are underrepresented as participants in these genetic studies [25,26]. Most genetic studies for depression prevention have been conducted in European populations abroad and included persons with mostly European ancestry in the United States [24,27]. Persons of European ancestry dominate all genetic studies (88%), with a small portion of people with Asian (6%), African American/Afro Caribbean (2.13%), Hispanic/Latino (1%), Mixed/other (0.67%), African (0.57%), and Native American (<0.5%) ancestry represented [25,26,28]. Studies show that persons who self-identify as European have mostly European ancestry, whereas persons who identify with an ethnic/minority group (i.e., Black/African American, Hispanic/Latino, Native/American), have traits of European, African and Native/American ancestry due to genetic admixture [29]. Thus, we must include those who self-identify with an ethnically diverse, non-European minority group to increase the ethnic diversity of the gene pool and enhance scientific study. 

In addition, most genetic studies have historically contained samples of women who are married [30,31,32], higher income professionals [28,33,34],, and older [29,30,34], thus not representative of younger subgroups of Black mothers who are often single and of lower socioeconomicstatus [9,35]. Evidence suggests that there is no single gene linked to depression [1]. Findings indicate that genetic risk variants may interact with demographics such as environmental and sociocultural factors and alter the risk of mental illness. Hence, samples with diverse demographic characteristics are needed to further examine the possibility that these interactions are occurring [1]. Women and minorities have been historically excluded from clinical research, and thus, including them has become a national health priority [1]. These government recommendations have been made based on strong evidence that lack of diversity with regard to race/ethnicity, gender, and socioeconomic and social status will decrease the generalizability of findings from these studies to unique groups such as Black/African American mothers. 

Diversity is needed in genetic studies for mental health disorders to ensure accurate assessment and prediction of risk, tailor effective treatments and interventions, reduce mental health inequalities, and promote health equity for underrepresented groups [25,26,27]. Different populations exhibit variations in genetic architecture that when compared and analyzed, provide critical information for understanding and predicting treatment responses and developing treatments tailored for each individual, thus contributing to precision medicine [22,36]. Including diverse populations in genetic research studies ensures that depression diagnosis, prevention, and treatment efforts will eventually be applicable to diverse populations of Americans. Efforts aimed at understanding the factors that lead to greater inclusion of ethnic/minority and majority groups of diverse socioeconomic backgrounds in genetic research are needed to enhance scientific discoveries for application to diverse high-risk, subgroups such as Black/African American mothers.

One factor known to impact participation in health-promoting behaviors, such as engagement in genetic research, is an individual’s unique beliefs about the behavior. According to the Theory of Planned Behavior (TPB), a person’s beliefs about a behavior impact their intention to engage in or perform that particular behavior [37,38]. Many researchers have used qualitative methods to identify the beliefs that impact the participation of aggregate samples of ethnic/minority women in genetic research for non-psychiatric conditions including genetic testing for sickle cell disease, hypertension, and human papillomavirus detection in African American women [38,39,40], breast cancer screening in African American and Hispanic women [41,42,43], and genetic biobanking in general for mostly female African American and White/Caucasian women [44,45]. A recent literature search revealed only one study in community-dwelling Black/African American adults who were mostly male (69.2%; 18/26) and with only four Black females (15.4%; 4/26), where qualitative methods were used to identify beliefs that impact participation in genetic testing for psychiatric disorders [46,47]. Other studies about psychiatric genetic testing have been conducted with U.S. medical professionals, and not community-dwelling adults [34,46]. No researchers have used qualitative methods to explore the beliefs of solely subgroups such as Black/African American mothers. We may be inclined to apply beliefs revealed by these aggregate samples of Black women and other groups regarding other forms of genetic testing towards psychiatric testing. However, according to behavior theory, beliefs are specific to the behavior and must be elicited from the population of interest [38]. 

Understanding the unique beliefs that underlie participation in genetic testing for psychiatric disorders from the perspective of Black/African American mothers is necessary to develop strategies to support their inclusion in these studies and ultimately, prevent depression in this at-risk group of mothers. The purpose of this analysis was to use the Theory of Planned Behavior to identify the most salient normative, behavioral, and control beliefs towards participation in genetic deoxyribonucleic acid (DNA) testing for the purpose of depression prevention and treatment in Black/African American mothers. This study also examined levels of depressive symptoms as well as determined the willingness of these mothers to participate in DNA testing. Based on the findings, future recommendations to increase the engagement and inclusion of Black/African American adult mothers in genetic research studies that may involve invasive biological sampling testing procedures for depression prevention and treatment are presented.

### Theoretical Background

The theory of planned behavior (TPB) served as the guiding framework for this study. The TPB postulates that the intention to perform a given behavior can be predicted from attitudes, subjective norms, and perceived behavioral control, and subsequently, intentions (i.e., motivational factors) are the antecedent of actual behavior [38,48]. Moreover, attitudes, subjective norms, and perceived behavioral control are determined by a set of salient normative, behavioral, and control beliefs about the given behavior. Attitudes refers to the evaluation of behavior as either favorable or unfavorable. Subjective norms refer to the perceived social pressure to perform the given behavior. Perceived behavioral control refers to the individual’s belief in their ability to perform the given behavior [38,48]. The TPB has been successfully used to elicit beliefs towards participation in mammography screening, vegetable consumption, hypertensive health care behavior, and a variety of other health behaviors in African American women [49,50,51]. However, TPB studies with a focus on psychiatric genetic testing and research are relatively limited [52]. 

## 2. Materials and Methods

### 2.1. Design

A descriptive, qualitative design was employed [53,54]. This current sample is a subset of a larger sample taken from data collected during a pilot study that examined beliefs about participation in salivary DNA sampling in 41 low-income or ethnic minority mothers from four different ethnic groups. Content analysis of questionnaire data that asked about salient beliefs took place and this larger analysis revealed the most salient beliefs from the perspective of that sample of multi-racial/ethnic mothers as a whole [53,54]. Racial/ethnic differences in salient beliefs were evident. This study therefore provides a separate, in-depth analysis of the responses solely of the Black/African American mothers of the original sample. 

### 2.2. Participants

In the original study, convenience, purposive sampling was used to obtain a sample of multi-racial/ethnic mothers (i.e., White/Caucasian, Black/African American, Hispanic/Latino) who would be able to detail the beliefs from the perspective of the target population [53,54]. The TPB recommends that salient beliefs be elicited from at least 30 mothers [38]. In the original study, a larger sample size of 41 mothers was recruited to obtain a larger representation of mothers from each racial background. The data from the 19 Black/African American mothers, who made up 46.3% of this original sample, were used for this analysis. Data saturation was not the goal in the larger pilot study. Data saturation, however, was reached with these 19 participants who reported strikingly similar sentiments despite the diversity of settings in which they were contacted. Prior research studies have similarly been successful at eliciting beliefs about genetic testing for cancer and psychiatric illnesses in general in Black/African Americans and women, reaching data saturation in samples of comparable sizes [43,47,55]. Women were included if they were of low income (<200% federal poverty line according to family size) [56,57,58] or racial/ethnic minority status [56] and had at least one child less than 18 years of age living with them. Mothers were excluded if they had a current diagnosis of depression or were being treated for depression, since this study was about prevention of depression. This study was also not about postpartum depression. Mothers who had children less than 1 year of age or mothers who were currently pregnant, therefore, were also excluded to avoid those who may have active symptoms associated with pregnancy or the postpartum period. Current research shows that the prevalence rates of depression are increasing among younger populations [59]. Hence, in the original study, mothers 46 years of age or older were excluded to increase the proportion of younger mothers in the sample. Mothers were also excluded if they were unable to read and understand English, since all instruments were written in the English language. 

### 2.3. Procedures and Recruitment

Recruitment started after the study was approved by the university’s Institutional Review Board (IRB). Mothers were recruited in four different urban cities in two different states at seven different community locations. Recruitment sites included a low-income housing complex, a social service agency, a daycare center, and three pediatric primary care practices. Flyers were given to the site owners/directors for display and distribution. At the social service agency, housing complex, and daycare center, those who inquired contacted the site owners/administrators, who organized a time for recruitment that took place weekly over a two-month period. Site owners/administrators served as champions and encouraged participation among the mothers they serviced. At these locations, screening and questionnaire completion took place on scheduled days in waiting areas and/or in rooms designated by the site owners/administrators. Children of participants were kept occupied with age appropriate toys and activities and/or had designated childcare aides provided by the center to allow for undisturbed questionnaire completion by mothers. The principle investigator, co-investigators, and undergraduate student research assistants who administered the questionnaires during recruitment were mostly Black/African American mothers. Women were approached in-person in waiting areas and were screened for eligibility in writing at the three pediatric offices and the dance school where subjects completed questionnaires. Written informed consent was obtained from those meeting study delimitations. All participants completed a demographic data sheet, a scale to measure levels of depressive symptoms, and an open-ended survey to elicit salient beliefs. Study investigators and research assistants were available in person to answer any questions that the mothers had. The women received a USD 10.00 Visa bank card as remuneration for participation immediately after the questionnaire packets were completed. 

### 2.4. Instruments

#### 2.4.1. The Centers for Epidemiologic Studies Depression (CES-D) Scale

The CES-D scale is a 20-item self-report instrument designed to measure current levels of depressive symptoms in the general population of U.S. adults [60]. Participants indicated on a 4-point summated rating scale (0 = situation occurred rarely or none of the time and 3 = most or all of the time or 5–7 days) the frequency and duration of times they have experienced certain situations or feelings. Four items (4, 8, 12, and 16) are reverse-scored and a total scale score ranging from 0 to 60 can be obtained. Evidence of content and construct validity and reliability was obtained during scale development [60]. Construct validity and reliability has been established in low-income and ethnic/minority mothers [9,11,12]. The Cronbach’s alpha reliability coefficient for internal consistency was 0.88 for the original sample in the larger study. For this subset of Black single mothers, the Cronbach’s alpha coefficient was 0.89.

#### 2.4.2. Salient Beliefs about DNA Sampling

An open-ended, free response questionnaire with nine items was developed based on the instructions specified in the TPB for eliciting salient beliefs [38,61]. Participants responded to questions that ask them to write in their behavioral, normative, and control beliefs about providing saliva samples for DNA analysis for the purpose of examining ways to prevent and treat depressive symptoms [61]. This questionnaire has been used to elicit salient beliefs about health behaviors in a variety of populations, including dietary supplementation in Black women [62], physical activity in pregnant women and parents [63,64], and prostate cancer screening in Black males [65]. 

#### 2.4.3. Genetic (DNA) Sampling Acceptability Questionnaire

In prior studies, Black/African Americans reported fears about harmful study procedures for psychiatric genetic testing [47] and Black women desired to have knowledge about what testing entailed for hereditary breast and ovarian cancer screening [42,43]. This 13-item questionnaire was therefore developed to provide knowledge about the procedures for salivary DNA testing. This questionnaire assesses participants’ willingness to perform the step-by-step sampling procedures as outlined in the instructions for providing DNA salivary samples in the insertion packet for the Oragene saliva DNA self-collection kit developed by DNA Genotek [66,67]. This test kit’s use has been tested in diverse populations and has found to be reliable, valid, and easy to use [66,67]. Numerous studies have also demonstrated adequate high-quality DNA yield by using the DNA Genotek saliva sampling methods that were useful for genetic analyses during scientific investigations [66], including identification of risk variants for depressive disorders [25,66]. There are nine questions that ask participants to respond “yes” or “no” to each of the steps for providing a sample. There are two questions that ask about procedures after the sample is collected, one question that asks about fears of genetic salivary testing, and one question about general willingness to participate. 

### 2.5. Data Analysis 

Descriptive statistics were used to summarize and describe the demographic data. These data were generated using IBM’s SPSS statistical computer program. An analysis of the responses from Black mothers was taken from a larger study’s multi-ethnic sample. The data were analyzed using qualitative methods involving coding and category formation [53,54]. A directed approach to content analysis was used to analyze responses from the questionnaire about salient beliefs [68]. As per this approach, codes and categories consistent with the theoretical constructs of the TPB (i.e., normative, behavioral, and control) were identified and used as the basis for initial coding schemes and categorization of the data [38]. Responses were entered into a 9 × 27 dimension table that was used as the basis for initial coding. Initial primary coding yielded 50 codes representing behavioral beliefs, 45 codes representing normative beliefs, and 24 codes representing control factors. During secondary coding, primary codes were collapsed, subsequently producing five categories for behavioral beliefs, seven for normative beliefs, and six representing control factors. For example, references to mothers, siblings, grandparents, and children were collapsed into the category labeled family members. Statements that referenced the desire to find out if they or others were depressed were collapsed into a category labeled diagnosis/detection. Categories were compared and ranked based on frequency of reporting of incidences of coding that represent the most salient beliefs about participation in testing. The percentages or frequency of codes and resulting categories for the total sample were reported as descriptive evidence of the theory [68]. The TPB was used as a guide, but did not constrain study findings, since codes/categories unrelated to the theory were also presented that included statements where subjects did not have a specific belief that matched a theoretical code/category (i.e., no disadvantages, no barriers, not sure). The categories were organized according to the TPB constructs. A frequency count was used to calculate the proportion of mothers who expressed a willingness to participate in genetic sampling and to calculate the proportion of the steps of the procedures for sampling that the mothers agreed to perform. Pearson product moment correlations were used to examine the relationship between depressive symptoms, educational level, income, age, and number of children.

### 2.6. Trustworthiness of the Data

To contribute to the trustworthiness of the qualitative data, site, source, and analyst/investigator triangulation was used to increase the credibility of the findings [53,54]. To reduce the effects of local factors that may introduce bias, mothers from seven different community sites, in two different states, comprising four different cities participated. In addition, a wide range of mothers at each community-based site participated, including mothers who utilized the services (i.e., clients, patients) and mothers who worked at each site (i.e., receptionist, childcare providers, medical assistants, food service workers). For analysis/investigator triangulation, both doctorally prepared investigators and undergraduate students participated in data collection. The analysis of the data was also performed by seven female doctorally prepared professors and three master’s prepared professor, along with two undergraduate student research assistants who independently analyzed the data and compared findings. Each have experience conducting research and working clinically with mothers who reside in under-resourced communities. The undergraduate research assistants were trained in data collection and analysis by the primary doctorally prepared investigator. All agreed on the final categories and themes.

## 3. Results

The participants consisted of 19 Black/African American mothers between the ages of 21 and 42 years (M = 31.06, SD = 6.11). All mothers indicated that they were born in the United States. Most indicated they were single having never been married (14/19, 73.7%), while the remainder were married (4/19, 21.1%) or divorced (1/19, 5.3%). Most of the mothers were of low-income (13/19, 68.4%), were employed full-time (11/19, 57.9%), and had a high school education only (14/19, 73.7%). Most mothers had one to two children (13/19, 68.4%) and 52.5% (11/19) had children less than 6 years old. Additional demographic information can be found in Table 1. Exactly 47.4% (9/19) of mothers scored greater than 16 on the CES-D scale, indicating clinically significant levels of depressive symptoms. Average depressive symptom levels were also abovethe cut-off of 16 that indicatesclinically significant levels of depressive symptoms that warrant psychiatric referral (M = 17.10, SD = 12.4) [60]. There was a positive relationship between educational levels and depressive symptoms, as mothers with higher levels of education reported higher levels of depressive symptoms (*r* = 0.470, *p* < 0.05). The relationships between depressive symptoms and income levels, age, number of children, and community location were not significant (*p* < 0.05). 

### 3.1. Most Salient Behavioral Beliefs 

#### 3.1.1. Advantages

The most salient advantage reported (6/19, 31.6%) was the belief that testing would help diagnose/detect depression in themselves (3/19) or in others (3/19). One mother stated, “You can find out if you may be depressed.” Other advantages mentioned included that testing would help find a cure or treatment for depression (5/19, 26.3%) as one mother stated, “to try and cure different symptoms in the world”. Mentioned once were beliefs that participating would help other people (1/19, 5.3%), would determine a genetic cause (1/19, 5.3%), and would provide an opportunity for more study on it (1/19, 5.3%). Three women believed that there were no advantages (15.8%) and four women were unsure of the advantages (21.1%). Additional statements of behavioral beliefs are found in Table 2.

#### 3.1.2. Disadvantages

The most salient disadvantage reported was a lack of certainty about how to respond/follow-up after testing results to treat, get help for, or prevent depression if diagnosed (4/19, 21.1%). One statement included, “After receiving the sample result some people may not get the help they need or know where to go to get help.” Many mothers reported that there were no disadvantages to participating (5/19, 26.3%). These statements included, “none”. Two mothers (10.5%) indicated a lack of trust or confidence in how the samples may be used or mishandled. Four mothers (21.1%) reported that they were unsure of the disadvantages, stating “I don’t know”. Additional statements are found in Table 2.

### 3.2. Most Salient Normative Beliefs

#### 3.2.1. Referents Who Approve

The most frequently mentioned referents were family members (9/16; 47.4%; mothers, sisters, children) and agencies or organizations (4/16, 21.1%; i.e., women’s group, counseling offices, law enforcement, government, agencies for employment, LGBT organizations) when asked who would approve of them providing saliva samples for DNA testing. Mothers reported less often that their significant others (2/19, 10.5%; boyfriends, husband), doctors (1/19, 5.3%), church associates (1/19, 5.3%; pastor), and no one (2/19, 10.5%) would approve. 

#### 3.2.2. Referents Who Disapprove

Many mothers indicated that no one (6/19; 31.6%;) or church associates (4/19; 21.1%; church friends, Quakers, church, someone in church) would disapprove of them providing samples for DNA testing. Other mothers reported that their family members (1/16; 18.8%; mother, sister, parents), friends (1/19, 5.3%), significant other (1/19, 5.3%), and work associates (1/19, 5.3%) would disapprove.

#### 3.2.3. Referents Who Would Participate

Mothers indicated that family members (6/19, 31.6%; cousins, aunts, mother, sisters, children), friends (5/19, 26.3%), and agencies/organizations (4/19, 21.1%) would participate. Other mothers indicated that church associates (1/16, 5.3%; Christians), work associates (1/16, 5.3%; coworkers, boss), and doctors (1/19, 5.3%) would participate. Five mothers (26.3%) indicated that no one would participate and two mothers (10.5%) indicated that they did not know who would participate. 

#### 3.2.4. Referents Who Would Not Participate

Mothers indicated that no one (5/19, 26.3%) would refuse to provide samples. Other mothers indicated that their significant other (4/19, 21.1%; boyfriend, husband), family member (1/19, 5.3%), church associates (2/19, 10.5%), work associates (1/19, 5.3%), and friends (1/19, 5.3%) would refuse to participate. One mother (1/19, 5.3%) indicated that everyone would refuse to participate. 

### 3.3. Most Salient Control Beliefs

#### Barriers and Facilitators

Mothers indicated that unpleasant or difficult testing procedures (7/19, 36.8) and lack of information about the purpose of testing (5/19, 26.3%) were barriers to testing. Mothers’ individual statements included, “the test is simple and not time-consuming”, “That it’s nasty”, and “Not understanding the research behind the saliva”. Mothers also indicated that the location (4/19, 21.1%) and timing/scheduling of testing (2/19, 10.52%), and availability of financial compensation (3/19, 15.8%) can be a barrier/facilitator. Statements referencing these included, “location of the place” and “Yes if I get paid”. One mother indicated that religious beliefs would be a barrier. Seven mothers (31.3%) indicated that there were no barriers and six mothers (18.8%) indicated that there were no facilitators of participation. Additional control belief statements are found in Table 3.

### 3.4. Willingness to Participate in Genetic (DNA) Salivary Sampling 

When asked if they were willing to participate, most of the Black mothers (17/19, 89.5%) responded “yes”, indicating a willingness to provide a sample of saliva for DNA testing when asked. Two mothers indicated that they would not provide saliva for DNA testing. One of these mothers who disagreed stated it was “not safe”. The other mother who disagreed stated that she would not do it because of “not being aware of all test that it’s used for”. Out of the nine steps of the procedures, 84.2% (16/19) of mothers agreed to perform all nine of the steps of the sampling process. Two mothers agreed to perform only one step and one mother agreed to perform only seven steps. 

## 4. Discussion

This is the first study to elicit the most salient behavioral, normative, and control beliefs about participating in genetic (DNA) testing for depression prevention and treatment from the sole perspective of Black/African American mothers. The willingness of mothers to provide samples of saliva for genetic testing and levels of depressive symptoms was also determined. The alarming proportion of Black mothers in the present sample reporting high levels of depressive symptoms (9/19; 47.4%) is consistent with the levels of symptoms reported in prior studies of Black/African American mothers [3,9,10,11,12,19]. These mothers could have undiagnosed clinical depression. Since none of these mothers are receiving treatment, these findings further emphasize the importance of the current inquiry and the crucial need to focus on inclusion of this vulnerable subgroup of mothers and other similar groups of women in genetic research that focuses on depression prevention, detection, and treatment. Levels of depressive symptoms did not vary by income level and women with higher educational levels had higher levels of depressive symptoms. Prior studies have similarly found no protective effect of socioeconomic status for the mental health of Black American adults [69,70].

### 4.1. Willingness

Most of this subgroup of Black/African American mothers are willing to participate in genetic salivary testing for depression prevention and treatment (17/19, 89.5%). These findings are consistent with prior research, where all Black and White adults were willing to participate in psychiatric genetic research when asked in one study [47] and Black women in another study were open to the idea of hereditary breast and ovarian cancer genetic testing [42]. Research shows that Black Americans have a stigma towards mental illness and distrust of researchers and research institutions that have historically abused Black/African American human subjects [71,72,73]. However, only two mothers mentioned issues of distrust related to how the information may be used. Clearly then, the majority of the mothers in this sample are not hindered by trust barriers traditionally held by aggregate samples of Black/African Americans. They are willing to participate, but perhaps outreach to these mothers has not occurred. Perhaps testing has not been made accessible by addressing the behavioral, normative, and control beliefs uncovered in this analysis that may serve as obstacles and facilitators of genetic salivary testing for depression prevention and treatment.

### 4.2. Behavioral Beliefs

Mothers reported more advantages to testing compared to disadvantages. Receipt of a diagnosis of depression for themselves or other mothers was mentioned most often as an advantage. This is not surprising since low-income mothers, especially those with young children, are less likely to receive referral and treatment for depressive symptoms to determine if clinical depression is present [21]. Black mothers also often do not seek out professional medical practitioners when they feel depressed [10,74]. Some Black women may endorse a cultural mandate to be a “Strong Black Woman”, causing them to suppress emotions, avoid vulnerability, and ignore or deny depressive symptoms or view them as a normal part of life [74,75,76]. Healthcare practitioners also often misdiagnose depression in Black women due to the variability in symptoms presentation that does not always match symptoms on widely used depression screening tools [77,78,79,80]. Healthcare to Black Americans may often be affected by discriminatory practices, leading to low-quality care and discontinuation of mental health treatment [79,81]. These Black/African American mothers may, therefore, welcome the possibility that depressive illness may be detected in themselves or other mothers through a biological sample without having to admit it themselves or receive an inappropriate diagnosis from a healthcare provider. Diagnosis is thus viewed as a benefit of psychiatric salivary genetic testing for these mothers. 

In prior studies, receipt of diagnosis for oneself or others was not reported by African American women as an advantage of genetic testing for nonpsychiatric disorders. For breast cancer genetic screening, a sample of mostly middle-aged (*n* = 50, M age = 43; range 21–65+; [42]) and older adult African American women (*n* = 21, 66% >45 years, range 21–65+; [43]) reported receipt of a diagnosis as a disadvantage that would invoke fear or distress. For HPV genetic testing for cervical cancer, receipt of a diagnosis was also reported by African American women (*n* = 81, M age 40.9 years, range 18–81) as a disadvantage that may cause fear and embarrassment [41]. Cancer is a known leading cause of death among lay persons and likely the reason that receipt of a diagnosis would not be viewed as advantageous, invoking fear for the women in the aforementioned samples. Diagnosis as a benefit was also not reported by African American adults (*n* = 35, M age = 57 years, range 26–77 years) for genetic testing for less lethal, sickle cell disease [39]. The receipt of a diagnosis was not reported at all by a sample of mostly male (*n* = 14) Black and White adults for psychiatric genetic testing in general (*n* = 18, M age = 42; SD = range 42–65) [47]. For psychiatric illnesses like depression, the possibility of receiving a diagnosis may be more welcome by women who are at higher risk and especially mothers who do not readily receive or access mental healthcare. The benefits of possibly finding a cure/treatment for depression for society was also a salient benefit for Black and White adults towards psychiatric genetic testing [47] and other ethnic groups such as Hispanic/Latinos for breast and ovarian cancer screening [82,83,84]. Overall, belief in the receipt of personal benefits may impact these mothers’ willingness to participate in genetic testing for depression in Black/African American mothers. 

The most salient disadvantage, uncertainty about how to respond to or follow-up and get help to treat or prevent depression if it is discovered, was also not reported by Black and White adults for psychiatric genetic testing [47]. However, lack of certainty/fear about how to respond to positive genetic test results for cancer screening was reported in aggregate samples of African Americans [42,43] and Hispanic women for breast cancer genetic screening [44]. Mothers at-risk lack access to treatment and Black women often do not seek treatment [10,21,75]. This may contribute to uncertainty about pathways for follow-up with positive results and may, therefore, impact their willingness to participate in salivary genetic testing for depression.

### 4.3. Normative Beliefs

Mothers named family members most often as referents who would approve of or also participate in testing. In prior studies for nonpsychiatric conditions, Black women similarly discussed family members (i.e., sisters, daughters, nieces, granddaughters, male relatives) as primary motivators for genetic testing for hereditary breast and ovarian cancer [42,43]. A sample of Latino adults viewed the provision of information for children available through genetic testing for breast cancer as a benefit [83]. However, in a sample of mostly male Black and White adults, great hesitancy about alerting family members about participation in psychiatric genetic research was reported [47]. Clearly for women who are primary caretakers of their families, and particularly these Black mothers who were mostly single heads of households [85], views and impacts of testing on family members would logically be most salient. These mothers mostly perceive that many family members would support their participation.

It is not surprising that mothers identified social service and government agencies second most often as those who would more likely approve and participate. Black mothers, who are often single and live in poverty, receive social and financial support services from these agencies and must meet agency guidelines for approval to receive these services [35,85,86]. Mothers may perceive that the agency’s approval/disapproval of their actions could be tied to the receipt of these resources and thus, their views would be salient. In prior studies, impact or views of agencies or organizations were not mentioned by affluent, highly educated Black women for genetic testing for breast and ovarian cancer research, nor by mostly male Black and White adults toward psychiatric genetic research [42,43,47]. For these mothers who are mostly of low-income and educational attainment, the agencies that frequently render support are salient on their minds as entities that would more likely support their participation rather than discourage them. 

Church associates were reported most often as those who would disapprove or not participate in testing. Prior studies show that religion is generally used as the main source of coping with depression for African Americans [18,87]. Religion was described, not as a barrier, but as a source of coping by affluent, highly educated Black women for positive genetic breast cancer screenings [43]. Black and White adults did not mention religion at all for genetic screening for psychiatric research [47]. However, in another report, religion was not a primary source of coping with depression for Black single mothers mostly of low-income [10]. Research shows that persons of lower income levels have lower participation in organizational religiosity compared to persons of higher income levels [88,89,90,91]. Clearly, this sample of mostly low-income Black mothers view religious associates as those who would more likely discourage rather than support participation. 

### 4.4. Control Beliefs

Overall, many mothers reported that there were no barriers, indicating that genetic testing was under their control (7/19; 36.8%). Since mothers were given a description of the testing procedures for providing a salivary sample, usual sentiments about fears of testing were not expressed. The most salient barriers mentioned reflected their feelings about how unpleasant or difficult the performance of the procedure may be. Sentiments about unpleasant procedural steps were similarly expressed by African American women about self-collected samples for HPV genetic screening and biomedical research participation in general [41,92,93]. When not informed about the steps in the procedures for genetic screening, the potential that study procedures would be harmful or painful was reported by Black and White adults toward psychiatric genetic research [47] and Black women for breast and ovarian cancer screening [42,43]. This fear of bodily harm and painful procedures was also expressed by Mexican women for breast cancer genetic screening [44] and Latinos for biomedical research participation in general [82]. No doubt knowing the steps of the procedure reduced the amount of sentiments about fear of harmful study procedures in this sample. However, as evidenced by the mothers’ responses, when the procedural steps were known, the perception of them as aversive or difficult to perform were viewed as a barrier to participation for over 30% of the Black mothers in this sample. 

The second most salient disadvantage mentioned by Black mothers (5/19, 26.3%) was a lack of information about the purpose of salivary genetic testing for depression. Lack of knowledge about genetics and genetic research in general was similarly expressed as a barrier to psychiatric genetic testing by Black adult [47]. Black women also reported lack of knowledge regarding hereditary testing for breast and ovarian cancer as a barrier of testing [42,43]. Discussions and information seeking about depression may not be common due to the stigma of mental illness in the Black community [71]. There is also a history of abuse of Black human subjects in research in the United States that may cause hesitation with information seeking or participation in genetic research [72,73]. It is not surprising then that mothers’ lack of knowledge about the purpose and reasons for testing is viewed as a barrier to participation by this sample of Black mothers. 

The facilitators of participation reported by this sample included things that would make participation more convenient such as the location and timing of testing and the availability of monetary compensation. In prior studies, Black women similarly reported transportation and personal finances as barriers to participation and recommended having testing locations within their communities, at close locations, and conducted on the weekends and evening and timing that does not interfere with their work schedules for breast and ovarian cancer screening [42,43]. Black adults discussed the desire for monetary compensation as an incentive and recommended the use of an outreach van in neighborhoods to increase participation in psychiatric genetic research [47]. Black mothers who are of low-income and single, as most in the present sample are, have multiple roles and responsibilities and often lack social support [19,35,42,86]. They may not have the resources to travel to testing sites outside of their communities and may need financial support to do so. It is not surprising then that accommodations that may address some of these potential barriers were mentioned as facilitators.

## 5. Conclusions/Implications

Researchers who are interested in recruiting high-risk subgroups of young to middle aged Black/African American mothers into genetic studies for depression prevention and treatment can capitalize on their high willingness to participate. Clearly, mothers want to participate yet various social determinants at multiple levels (i.e., such as normative influences, lack of knowledge, situational barriers, inconvenient locations, times, financial constraints) may create barriers that impact participation and must be addressed [94]. To reduce barriers, researchers can employ community-based participatory research (CBPR) methods to facilitate participation. A CBPR approach allows for outreach to these communities and facilitation of open, ongoing discussions between researchers and community residents in a spirit of collaboration and shared ownership of the research priorities, benefits, and outcomes [95]. Open discussions allow for the identification of barriers and facilitators so that strategies to address them can be utilized. The effectiveness of this approach for encouraging health promoting behavior within ethnic/minority populations is well documented for many health-promoting behaviors [44,79,96,97]. To encourage researchers to use a CBPR approach, institutions/foundations that provide financial support for research can make the use of this method a requirement for the receipt of research funding. A recent review recommended that diversity in genomic research be incentivized by prioritizing funding and publishing for researchers that utilize samples of groups that are diverse and/or under-represented such as Black/African American mothers [98]. 

The identified advantages, disadvantages, normative referents, barriers, and facilitators of testing from the perspective of this subgroup of mothers can be used to inform the design of future genetic studies that target this subpopulation of mothers. Based on the responses, researchers can conduct educational sessions within their communities or make sure education is part of the informed consent process. The personal benefits of testing, such as being able to detect, treat, cure, or prevent depression in the future, can be stressed. Misconceptions about immediate diagnostic and curative benefits can be clarified. Mothers can also be instructed about possible results of testing and locations for evaluation and treatment should they receive information from testing that requires follow-up. Counseling can also be offered prior to testing and after testing so that all of the mothers’ questions and concerns can be addressed. 

Some mothers viewed the procedures for salivary testing aversively or as difficult to perform. Research shows that ethnic/minority women and mothers may prefer certain biological testing methods over others [41,92,99]. There are many methods for collecting DNA samples [100,101,102]. Procedures for testing can be discussed during educational sessions and researchers can agree to use the procedures that community members prefer. Family members who are supportive of participation can also be invited to these discussions. 

In addition, researchers can collaborate with the government and social service agencies that these mothers frequent and perhaps develop collaborative research proposals to conduct the research in these convenient community locations where the mothers reside, since many of these mothers view these agencies as supportive of their participation. Collaborative discussions along with the willingness of researchers to make the needs of these mothers a priority may foster trust, breaking barriers created by known abuses of Black/African American human subject research participants [94,95]. Efforts to have researchers who are familiar with or are of similar backgrounds as participants may also lessen issues of trust related to participation in clinical research. The national institute of mental health recognizes the need for a diverse mental health research workforce that is crucial for eliminating mental health disparities [103]. Diverse researchers may be more successful at encouraging participation in genetic research among participants of similar, underrepresented backgrounds and may provide unique and broadened perspectives for addressing research questions and eliminating health disparities among diverse subgroups such as Black/African American mothers [103].

Though many Black/African Americans use religion as a means of coping with depression, the Black/African American mothers in this sample generally felt religious associates would not support genetic testing for depression prevention and treatment. The role of religious views among Black/African American mothers and its relation to genetic testing, depression prevention, and treatment need further exploration using qualitative methods of research 

### Limitations

A sample of convenience is also inherently biased due to self-selection into this study. Most recruitment sites were located in low-income communities, so the sample was comprised of mostly low-income Black mothers. To obtain adequate representation of Black/mothers of higher socioeconomic status, locations in higher income communities would need to be targeted. The sample size was small, less than 30 participants, as recommended by the TPB for eliciting salient beliefs [38]. Results should be interpreted in light of this fact. These results may also only be applicable to those who reside in urban communities, since the locations of the recruitment sites were in inner city neighborhoods. In addition, use of incentives can produce non-response bias that may inherently impact the results of this study in some indeterminant way [104,105,106].

## Figures and Tables

**Table 1:** Frequency distribution of selected demographic variables (*n* = 19).

Characteristic	*n*	Percentage
Ages		
	17	89.47%
Mean	31.06	
Stand Dev	6.11	
Range	21–42	
20–29	8	47.06%
30–39	7	41.18%
40–45	2	11.76%
Marital Status	19	100%
Single/Never Married	14	73.68%
Married	4	21.05%
Divorced	1	5.26%
Head of Household	19	100%
Yes	15	78.95%
No	4	21.05%
Number of Children	19	100.00%
2 or fewer	13	68.42%
3–4 children	5	26.32%
5–6 children	1	5.26%
Child Age Less than 18 years	19	
Less than 6 years	10	52.6%
6 years or older	9	47.4%
Education (highest grade completed)	19	100.00%
High School	14	73.68%
Technical School	1	5.26%
Two-Year College	2	10.54%
Four-Year College	1	5.26%
Masters Program	1	5.26%
Employment	19	100.00%
Full-Time	11	57.89%
Part-Time	3	15.79%
Unemployed	5	26.32%
Income	18	100.00%
Less than USD 5000	2	11.11%
Between USD 5000 and 20,000	6	33.33%
Between USD 20,000 and 30,000	2	11.11%
Between USD 30,000 and 40,000	1	5.55%
Between USD 40,000 and 50,000	2	11.11%
Between USD 50,000 and 60,000	3	16.67%
Greater than USD 70,000	2	11.11%

**Table 2:** Salient behavioral beliefs (*n* = 19).

Salient Behavioral Beliefs	*n*	Quotations
Advantages		
Diagnosis or detection of depression	6	“See what level or race has the most depressive symptoms”
Treatment/cure for depression	5	“Detect natural ways to combat depression” “To try in cure different symptoms in the world “If it helps finds cures I’m for it”
Helping others	1	“It can help other people”
No Advantages	3	“none”
Not Sure of Advantages	4	“I don’t know”
Disadvantages		
Uncertainty about follow-up and results	4	“I wouldn’t know unless I try itonly bad I can see come out of it is me not having depression”
Trust	2	“not being aware of all test that its used for.”
No disadvantages	5	n/a
Not sure of disadvantages	4	“I don’t really know”

**Table 3:** Salient control beliefs (*n* = 19).

Salient Control Beliefs	*n*	Quotations
Procedures: Unpleasant/Difficulty/Ease	7	“Putting different saliva together to get a surge”
		“it’s unclean and non-hygienic”
Information/Explanations of study purpose and procedures	5	“I think why use saliva instead of a questionnaire”
		“A lot more questions about what exactly is going to be able to be proven and fixed about it”
		“I do not know what would help with a DNA test”
Location	4	“If the location isn’t easily accessible”
		“Mobile communicators of the information”
Time/Scheduling	2	“Convenient time and location”
		“Long work hours”
Compensation	3	“For pay”
Religion	1	“Someone heavy into their religion”
No barriers	7	“None”
No facilitators	6	“None”
